# Kinetic properties and small-molecule inhibition of human myosin-6

**DOI:** 10.1016/j.febslet.2012.07.014

**Published:** 2012-09-21

**Authors:** Sarah M. Heissler, Jayashankar Selvadurai, Lisa M. Bond, Roman Fedorov, John Kendrick-Jones, Folma Buss, Dietmar J. Manstein

**Affiliations:** a Institute for Biophysical Chemistry, OE4350, Hannover Medical School, 30623 Hannover, Germany; b Research Division for Structural Analysis, OE8830, Hannover Medical School, 30623 Hannover, Germany; c Cambridge Institute for Medical Research, University of Cambridge, Department of Clinical Biochemistry, Wellcome Trust/MRC Building, Cambridge CB2 0XY, UK; d MRC Laboratory of Molecular Biology, Hills Road, Cambridge CB2 2QH, UK

**Keywords:** Actin myosin, Kinetics, Inhibition, Allostery

## Abstract

Myosin-6 is an actin-based motor protein that moves its cargo towards the minus-end of actin filaments. Mutations in the gene encoding the myosin-6 heavy chain and changes in the cellular abundance of the protein have been linked to hypertrophic cardiomyopathy, neurodegenerative diseases, and cancer. Here, we present a detailed kinetic characterization of the human myosin-6 motor domain, describe the effect of 2,4,6-triiodophenol on the interaction of myosin-6 with F-actin and nucleotides, and show how addition of the drug reduces the number of myosin-6-dependent vesicle fusion events at the plasma membrane during constitutive secretion.

## Highlights

► First description of the production and detailed functional characterization of a human myosin-6 motor. ► Identification of halogenated phenols as inhibitors of myosin-6 activity. *►* In vitro and in vivo characterization of the effect of tri-iodophenole on myosin-6 function.

## 1 Introduction

Eukaryotic cells depend on dynamic, spatio–temporal rearrangements of the cytoskeleton mediated by the myosin superfamily of actin-based molecular motors [1]. In contrast to all other myosins studied so far, myosin-6 moves towards the pointed end of actin filaments [2]. This property originates from a structurally unique, class-specific insertion within the motor domain that redirects the lever arm by approximately 120° [2,3]. Myosin-6 has only been detected in higher eukaryotic cells; in humans it is encoded by a single gene (*MYO6*) [4]. The protein participates in multiple physiological processes including cell migration, the intracellular transport of organelles and vesicles, endocytosis, and secretion [5–10]. Moreover, myosin-6 is receiving much interest as a therapeutic target since it is implicated in the onset and progression of various diseases such as astrogliosis and various forms of cancer [5,7,11,12]. Therefore, selective myosin-6 inhibitors are postulated to have great medicinal relevance.

Despite its importance, the exact kinetic and functional properties of human myosin-6 have not been studied in detail and previously identified small-molecule inhibitors of myosin motor activity display no or only weak potency against myosin-6 [13–15]. Here, we report the kinetic properties of this unconventional myosin motor and describe the effect of the poly-iodinated phenol derivative 2,4,6-triiodophenol (TIP) on the ATPase activity and cellular function of human myosin-6. TIP, also known as Bobel-24 or AM-24, has been described as a nonsteroid anti-inflammatory molecule due to its inhibitory effect on leukotriene B4 (LTB4) synthesis [16]. Additionally, TIP acts as a thyroid hormone disrupting chemical and displays potent anti-cryptosporidial activity [17,18]. Our results show that TIP, when used in low micromolar concentrations, is targeted to myosin-6. Binding of TIP to myosin-6 results in reduced motor activity and functional impairment of the protein’s physiological role during the final stages of the secretory pathways.

## 2 Materials and methods

### 2.1 Construction of baculovirus transfer vectors and preparation of recombinant proteins

A protein engineering approach was chosen to fuse amino acids 1-816 of human myosin-6 motor domain to an artificial lever derived from *Dictyostelium discoideum* α-actinin [19]. The myosin motor domain construct M6-2R was overproduced in the baculovirus/Sf9-system and purification was facilitated by a C-terminal octahistidin-tag as reported previously [20]. Rabbit skeletal muscle actin was prepared as described by Lehrer and Kerwar [21] and labeled with pyrene iodoacetamide as described by Criddle et al. [22].

### 2.2 Kinetic and binding studies

Steady–state kinetics were performed at 25 °C with the NADH-coupled assay as described earlier [20]. Data from the inhibition of M6-2R actin-activated ATPase by TIP were fitted by the function:

(1) $$y=\left(y_{\text{max}}-y_{\text{min}}\right)\left[\left(1-P_{\text{low}}\right)/\left(1+{\left(x/{\text{IC}}_{50(\text{high})}\right)}^{\text{H}}\right)+P_{\text{low}}/\left(1+{\left(x/{\text{IC}}_{50(\text{low})}\right)}^{\text{H}}\right)\right]+y_{\text{min}}$$

with *y*, actin-activated ATPase activity; *x*, inhibitor concentration; *P*_low_, proportion of low-affinity binding sites; IC_50(low)_, IC_50_ of lower-affinity binding site; IC_50(high)_, IC_50_ of higher affinity binding site.

**Fig. 1:**
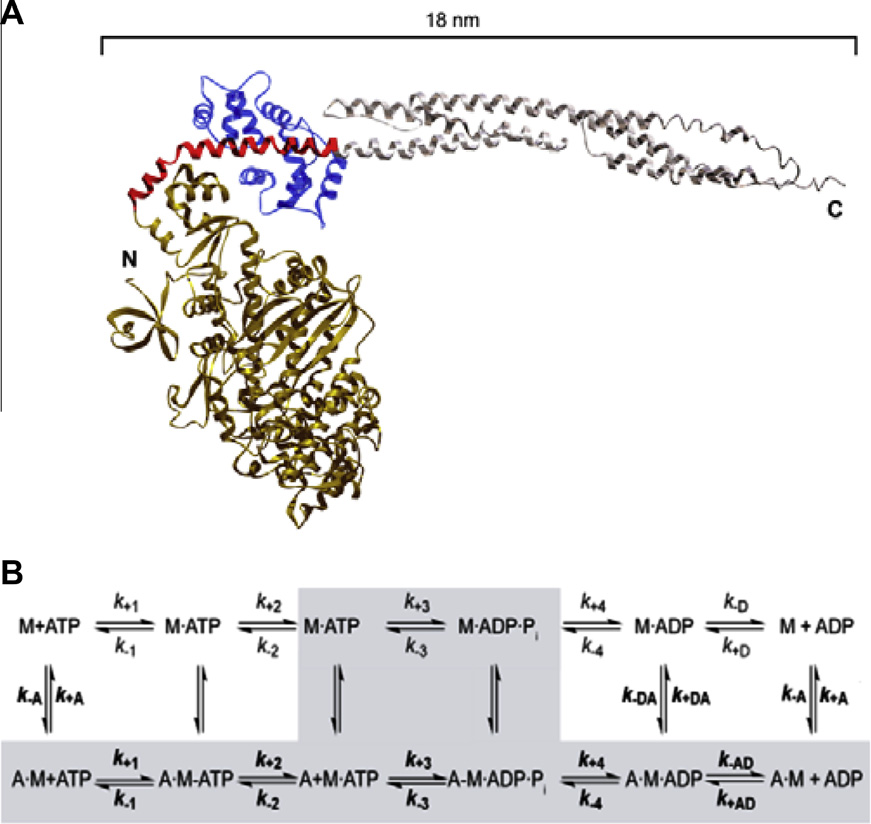
Model of the myosin-6 fusion construct with artificial lever arm and basic reaction scheme for the myosin-6 ATPase cycle in the absence and presence of F-actin. (A) Model of the engineered construct based on the experimental structures of the myosin-6 motor domain(pdb-code: 2BKI) and neck (pdb-code: 3GN4) regions fused to an artificial lever arm derived from α-actinin repeats 1 and 2 (PDB-code: 1G8X). The myosin motor domain is shown brass-colored, calmodulin in blue, insert-2 in red, and α-actinin in grey. (B) The actin-dissociated pathway including the steps ATP binding, ATP hydrolysis, and product release is shown in the upper line. The equivalent steps for the actin-associated pathway are depicted in the lower line. The predominant flux through the reaction pathway is highlighted in grey. Kinetic parameters in the absence and presence of F-actin are distinguished by using regular (*k*_+1_, *K*_1_) versus bold (***k*_+1_**, ***K*_1_**) type; subscript A and D refer to actin (***K*_A_**) and ADP (*K*_D_), respectively. M refers to myosin and *P*_i_ to inorganic phosphate.

Stopped-flow techniques were employed to study the interaction of myosin with nucleotides and F-actin. Stopped-flow measurements were conducted at 20 °C with a Hi-tech Scientific SF-61SX2 stopped-flow system equipped with a 75 W mercury–xenon arc lamp in MOPS buffer [20 mM MOPS (pH 7.0), 100 mM KCl] [20]. Magnesium concentrations were adjusted by supplementing MgCl_2_ to the MOPS buffer. The concentration of free Mg^2+^ ions was calculated using Maxchelator software as (http://www.stanford.edu/~cpatton/webmaxcS.htm). Actin-binding assays in the presence and absence of ADP were performed at a constant [myosin]:[pyrene–actin] ratio of 1:5. Data storage and initial fitting were performed using the software Kinetic Studio 1.08 (TgK Scientific). The reactant concentrations stated throughout the text are those after 1:1 mixing in the stopped-flow spectrophotometer. Analysis of kinetic data was accomplished using the same basic models that were developed to describe the kinetic behavior of rabbit fast skeletal muscle myosins and other myosins [23–25]. Consistent with these models, kinetic parameters for the interaction of M6-2R with nucleotide and F-actin were analyzed in terms of the kinetic model shown in Fig. 1 [26].

Microscale thermophoresis (MST) was conducted with the Nanolith NT.015 instrument (NanoTemper Technologies, Munich) in buffer containing 25 mM HEPES (pH 7.5), 100 mM KCl, 5 mM MgCl_2_ and 1 mM DTT [27]. Human myosin-6 was labeled for detection in the MST experiments with the red fluorescent dye NT-647 (NanoTemper Technologies, Munich).

### 2.3 Myosin motor activity

Motor activity was determined by means of an in vitro motility assay [28]. The assay buffer used contained 25 mM imidazole (pH 7.4), 25 mM KCl, 1 mM MgCl_2_, 1 mM EGTA, 4 mM ATP and an oxygen scavenging system consisting of 0.1 mg/ml glucose oxidase, 0.02 mg/ml catalase, and 5 mg/ml glucose. Measurements were performed with an Olympus IX70 microscope, 40⨯ 1.30 Apo oil objective, Chroma HQ Filter set 41002, and a Hamamatsu Orca-R2 CCD-camera at a constant temperature of 30 °C. The velocity of individual actin filaments sliding was tracked with the help of the program DiaTrack 3.01 (Semasopht, Switzerland). Data analysis was performed with Origin 8.0 (OriginLab, USA).

### 2.4 Molecular modeling

Homology modeling of the human myosin-6 motor domain was performed based on the X-ray crystal structure of porcine homologue (PDB id: 2v26) using the program MODELLER [29]. Both motor domains share 97% sequence identity. Missing loops were built using the LOOPY module of Arp/Warp [30]. Blind and targeted docking was performed using AutoDockVina[31].

### 2.5 Live cell imaging

Live-cell TIRF microscopy of secretion events using a mutant FKBP protein construct (F36 M) including a green fluorescent protein (eGFP) tag, siRNA knockdown of myosin-6, and computer-aided vesicle tracking were performed according to Bond et al. [32].

## 3 Results

Our study consists of three sections. First, we report the detailed kinetic characterization of the human myosin-6 motor domain construct M6-2R. The second section describes in vitro studies describing the interaction between TIP and the myosin-6 motor domain and in the third section myosin-6 inhibition by TIP in live cells is reported.

### 3.1 Functional characterization of the human myosin-6 motor domain

To gain information about the functional behavior of human myosin-6, we examined the motile activity and performed a detailed characterization of the motor domain construct M6-2R (Fig. 1A). This construct contains the myosin-6 motor domain, a unique insert that is essential for (−) end directionality and its associated calmodulin, and an α-actinin sequence acting as an artificial lever arm [19]. Artificial lever arms were used to study the motor function of a wide range of conventional and unconventional myosins. The resulting fusion proteins are constitutively active and display kinetic and mechanical properties that are comparable to wild-type motor domain or S1-like constructs [20,33–37]. Myosin-6 motor domain constructs with fused artificial lever arms at several positions, retaining varying amounts of native structure were shown to display robust processive stepping [38,39].

**Fig. 2:**
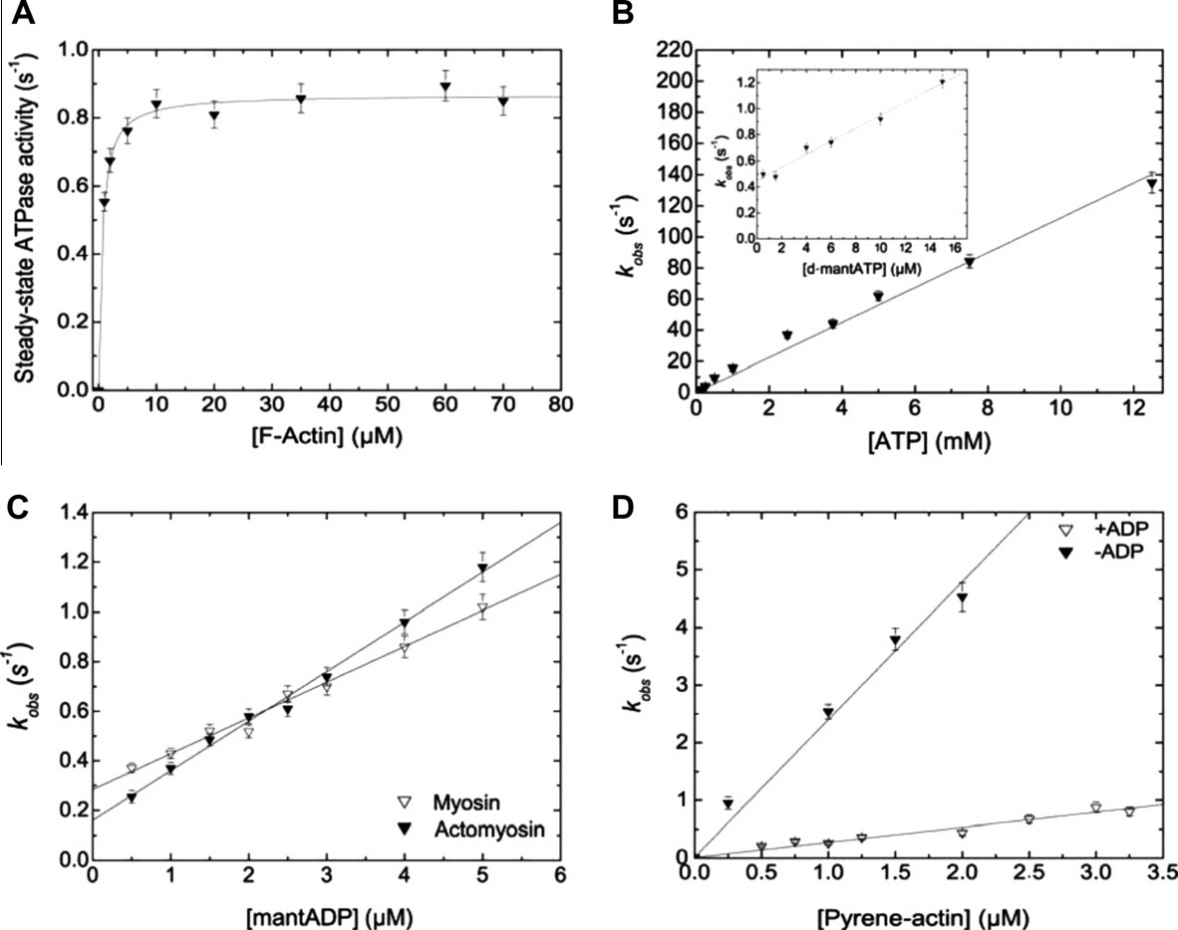
Kinetic characterization of the human myosin-6 motor domain. (A) Steady-state ATPase activity in the presence of F-actin. The parameters derived from the hyperbolic fit are 0.87 ± 0.01 s^−1^ and 0.58 ± 0.07 μM for *k*_cat_ and *K*_app_, respectively. (B) Interaction of acto•M6-2R and M6-2R with ATP. The observed rate constants (k_obs_) for ATP binding to the complex formed by pyrene–actin and M6-2R show a linear dependence on [ATP] in the range 0.1–13 μM. The slope of the best-fit line defines the second order binding rate constant ***K*_1_*k*_+2_** as 0.011 ± 0.0003 μM^−1^ s^−1^. Inset: Binding of d-mantATP to M6-2R. The observed rate constants display a linear dependence on [d-mantATP] in the range 0.5–15 μM. The slope defined the second order binding rate constant for d-mantATP binding to M6-2R as *K*_1_*k*_+2_ = 0.05 ± 0.01 μM^−1^ s^−1^. The first order rate constant for ATP dissociation from M6-2R *k*_−2_ is defined by the intercept as 0.45 ± 0.01 s^−1^. (C) Binding of d-mantADP to acto•M6-2R and M6-2R. Linear dependences of the observed rate constants are observed in the range 0.5–5 μM mantADP. The resulting slopes define the apparent second order rate constants for ADP binding to M6-2R and acto•M6-2R as *k*_+D_ = 0.14 ± 0.01 μM^−1^ s^−1^ and ***k*_+AD_ **= 0.20 ± 0.01 μM^−1^ s^−1^, respectively. The corresponding dissociation rate constants are defined by the intercepts as *k*_−D_ = 0.28 ± 0.01 s^−1^ and ***k*_−AD_ **= 0.16 ± 0.02 s^−1^. (D) Modulation of the interaction between M6-2R and pyrene–actin by ADP. The rate constants observed in the absence and presence of saturating concentrations of ADP are linear dependent on [pyrene–actin]. The slopes of the best-fit lines show pronounced differences and define the apparent second order rate constants for actin binding in the presence and absence of ADP as ***k*_+A_ **= 2.4 ± 0.08 μM^−1^ s^−1^ and ***k*_+DA_ **= 0.26 ± 0.01 μM^−1^ s^−1^.

Analysis of the velocity of fluorescent-labeled actin-filaments over a M6-2R-decorated glass-surface gives a mean velocity of ∼20 nm s^−1^ (data not shown). This confirms the functional competence of the recombinant myosin motor domain construct but the slow rate of movement hinders a more detailed characterization of motor function. In contrast, the turnover of ATP displayed by the construct is well-suitable for a more detailed characterization. Fig. 1B depicts the basic reaction scheme for the myosin ATPase cycle and defines the nomenclature for the rate and equilibrium constants used during the following discussion of the kinetic properties of human myosin-6. The addition of saturating concentrations of F-actin increases the steady-state turnover of ATP by M6-2R 24-fold, from 0.04 ± 0.004 s^−1^ (*k*_basal_) to 0.87 ± 0.01 s^−1^ (*k*_cat_). Half-saturation of activation is reached at an F-actin concentration of 0.58 ± 0.07 μM (*K*_app_) (Table 1 and Fig. 2A).

As depicted in Fig. 2B, the interaction of M6-2R and acto·M6-2R with the substrates ATP or 2′- deoxy- 3′- O- (*N*′- methylanthraniloyl)adenosine- 5′- O- triphosphate (d-mantATP) show a linear dependence in the range 0 to 15 μM d-mantATP. This yields apparent second order rate constants for ATP binding to M6-2R (*K*_1_*k*_+2_ = 0.05 ± 0.01 μM^−1^ s^−1^) and acto·M6-2R (***K*_1_*k*_+2_ **= 0.011 ± 0.0003 μM^−1^ s^−1^). Titration of M6-2R and acto•M6-2R with mantADP in the range 0 to 5 μM gives linear concentration dependences, with the slopes corresponding to the second-order rate constants for ADP binding to M6-2R (*k*_+D_ = 0.14 ± 0.01 μM^−1^ s^−1^) and acto•M6-2R (***k*_+AD_ **= 0.20 ± 0.01 μM^−1^ s^−1^). Dissociation rate constants *k*_−D_ = 0.23 ± 0.01 s^−1^ and ***k*_−AD_ **= 0.16 ± 0.02 s^−1^ were derived from the corresponding ordinate intersections (Fig. 2C). Similar dissociation rate constants were obtained from chase experiments, where we followed the dissociation of mantADP after the addition of a large excess of ATP (Table 1).

The rate of actin binding was measured following the exponential decrease in pyrene fluorescence observed on binding of an excess of pyrene–actin to M6-2R. The observed rate constants display a linear dependence in the F-actin concentration range studied (Fig. 2D). The slope of the plot defines the second-order rate constants of pyrene–actin binding ***k*_+A_** to 2.4 ± 0.08 μM^−1^ s^−1^. Similarly, a linear dependence of the observed rate constant on the concentration of F-actin was observed in the presence of saturating concentrations of ADP (250 μM). Here, the linear fit defines the second order rate constants of actin binding ***k*_+DA_** to 0.26 ± 0.01 μM^−1^ s^−1^. Direct measurements of the rate of M6-2R release from F-actin gave first order rate constants of ∼0.014 s^−1^ for ***k*_−A_** and ∼0.01 s^−1^ for ***k*_−DA_**. A comparison of the kinetic properties determined for our human myosin-6 construct with those measured previously for porcine myosin-6 wildtype and TEDS-site mutant constructs is shown in Table 1.

### 3.2 Inhibition of myosin-6 by TIP

Our initial search for selective small molecule inhibitors of myosin-6 motor activity was guided by results previously obtained with a congeneric series of halogenated pseudilins [13,15,40,41]. The low affinity of halogenated pseudilins for myosin-6 observed in these studies in combination with spatial constrains imposed by the allosteric pocket in myosin-6, guided us to the use of smaller compounds lacking either the phenyl or pyrrol moiety of the halogenated pseudilins. To identify suitable compounds, we used both computational simulation of the candidate ligands binding to the human myosin-6 motor domain and experimental binding studies.

We generated a homology model based on the crystal structure of porcine myosin-6 in the pre-powerstroke state (PDB id: 2v26) for the molecular docking studies. Human and porcine myosin-6 motor domains share 97% sequence identity. Initial blind docking with 2,4,6-tribromophenol, 3,4,5-trimethoxyphenol, pentabromophenol, and TIP gave top-ranked docking poses with the compounds either bound to the blebbistatin or halogenated pseudilin binding sites [40,42]. The predicted free binding energies obtained from targeted docking to both sites are in the range from −5.4 to −7.6 kcal/mol. The top-ranked docking poses for TIP to both sites have predicted free energies of binding *ΔG*_bind,free_ in the range from −5.4 to −5.8 kcal/mol. Despite docking scores in the lower range, microscale thermophoresis (MST) identified TIP as the highest affinity binders amongst the compounds tested (data not shown).

**Fig. 3:**
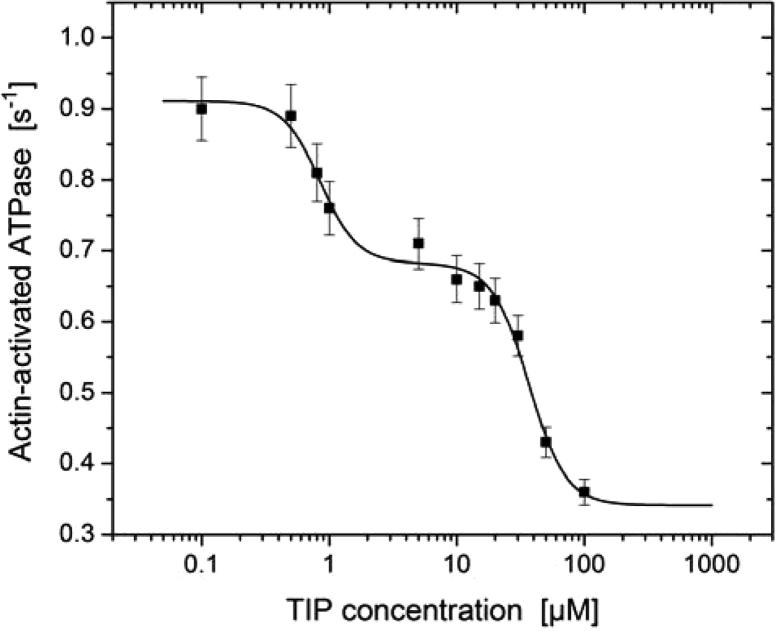
Inhibition of acto•M6-2R by TIP. The semilogarithmic plot shows the concentration dependence of the inhibition of M6-2R actin-activated ATPase activity by TIP with *K*_i1_ = 0.8 ± 0.5 μM and *K*_i2_ = 37 ± 2.6 μM. The maximal and minimal ATPase measured per motor domain (*y*_max_, *y*_min_) correspond to 0.91 and 0.34 s^−1^, respectively. The contribution of the lower affinity site to the inhibition corresponds to 63% (*P*_low_). The Hill coefficient (H) was set to 3. Error bars represent standard deviations from at least three determinations of each data point.

To obtain direct information about the effect of TIP on the catalytic activity of M6-2R, we performed actin-activated ATPase assays in the presence of increasing concentrations of TIP. The resulting data show an up to threefold inhibition of the maximum actin-activated ATPase activity and display a biphasic behavior, which can be explained by the presence of two independent binding sites (*K*_i1_ = 0.8 ± 0.5 μM and *K*_i2_ = 37 ± 2.6 μM) contributing 37% and 63% to the inhibition (Fig. 3). The actin-activated ATPase activities of *Dd* myosin-1D, human nonmuscle myosin-2C, and porcine β-cardiac myosin are not affected by the presence of up to 50 μM TIP (data not shown).

### 3.3 Live cell studies

**Fig. 4:**
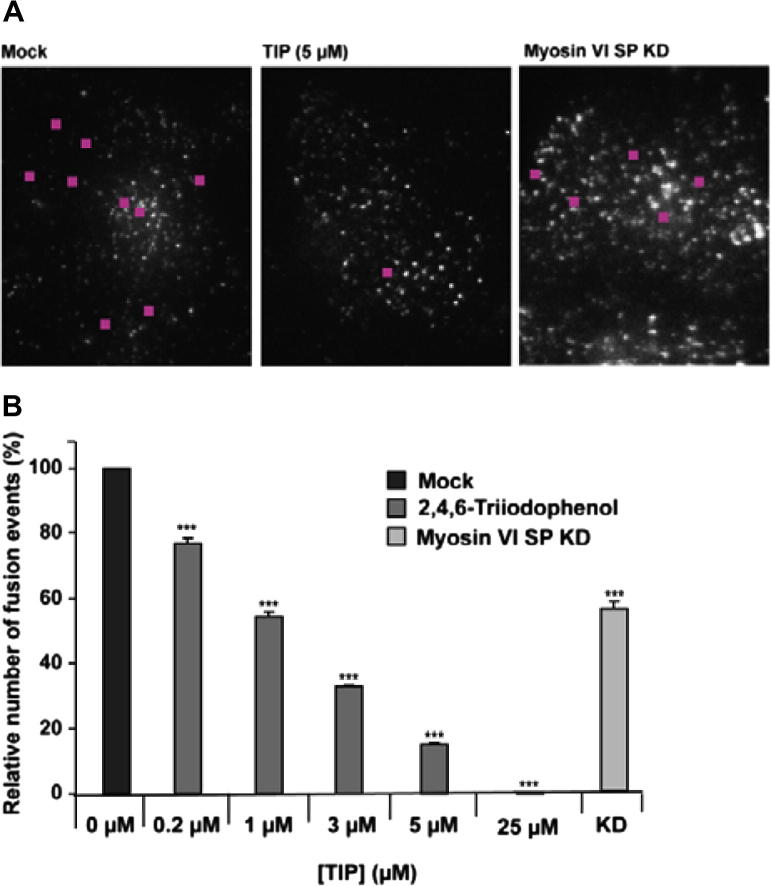
Inhibition of transport processes involved in secretion by TIP. (A) Sample images of the TIRF field at the base of a mock cell (left panel), a cell treated with 5 μM TIP (central panel), and a cell in which myosin-6 has been knocked down by siRNA transfection (right panel). Pink squares mark the location of vesicle fusion events over the course of a 5 s movie. (B) Dose-response diagram relating the relative number of fusion events to TIP concentration. This type of analysis indicates that TIP inhibits myosin-6 dependent processes with an IC_50_ value of 1.6 ± 0.6 μM. At the start of each experiment, cells were treated with the releasing ligand AP21998 to trigger secretion and simultaneously with a specific concentration of inhibitor (0–25 μM TIP). Ten cells were imaged in two-cell pairs 25–60 min after addition of TIP. Each data point represents the normalized sum of fusion events observed for 5 intervals of 5 min duration. The total number of fusion events observed in the absence of inhibitor was >1300 during the observation interval. Averages from 3 to 4 independent measurements are shown for each data point.

To probe the physiological consequences of TIP binding to myosin-6, we employed an evanescent-field microscopy-based secretion assay probing the final stages of the secretory pathway in a HeLa cell line that stably produces an eGFP-tagged mutant FKBP protein-based reporter construct [43]. Using this assay, it was shown that siRNA-mediated knockdown of myosin-6 reduces constitutive secretion levels by lowering the total number of secretory fusion events at the plasma membrane [32]. Similarly, TIP-treatment of HeLa cells also inhibits fusion events at the plasma membrane in a dose-dependent manner (Fig. 4). The 46% decrease in the number of fusion events after treatment of cells with 1 μM TIP is comparable to the 42% decrease in fusion events seen after a myosin-6 knockdown with a SMARTpool collection of four independent siRNA primers [32]. Plotting the resulting dose-response data indicates an IC_50_ value of 1.6 ± 0.6 μM for TIP (Fig. 4B).

## 4 Discussion

The results of our characterization of the motor domain construct M6-2R and its interaction with F-actin and nucleotides are summarized in Table 1 together with previously determined values for murine and porcine myosin-6 constructs. Despite 10-fold changes in individual rate constants, the kinetic properties of human myosin-6 show an overall resemblance to those determined for porcine and murine myosin-6 [25,44]. The observed values are all well within the range reported previously for the kinetic properties of different porcine myosin-6 constructs [2,25]. Coupling between the actin and nucleotide binding sites differs less than threefold, as indicated by the values measured for *k*_cat_/*K*_app_ and ***K*_AD_**/*K*_D_. Mammalian myosin-6 orthologs appear to share a slow rate of ATP binding to the rigor complex (***K*_1_*k*_+2_** = 0.011 μM^−1^ s^−1^). However, given that normal intracellular ATP levels are in the millimolar range, ADP release from actomyosin (***k*_−AD_**) appears to be the common rate limiting step under physiological conditions (0.5–5 s^−1^). Our results are thus consistent with a previously described physiological role for human myosin-6 serving as a motor involved in anchoring and tension generation in the final stages of the fusion of secretory vesicles with the plasma membrane [32].

Loss of myosin-6 function after siRNA KD causes a decrease in the number of secretory vesicles fusing with the plasma membrane and an increase in the number of vesicles that appear to be docked close to or tethered at the plasma membrane. Detailed analysis of the role of myosin-6 and its binding partner optineurin in this process has revealed a role for myosin-6 in fusion pore formation between secretory vesicles and the plasma membrane [32]. Small, drug-like effector molecules are useful tools that can be employed to dissect the role of the target protein in vivo and in vitro. Earlier work using a congeneric series of halogenated pseudilins [13,15,41], led us to choose a phenolic effector scaffold as lead to specifically target myosin-6. The initial molecular docking studies suggest possible binding sites for this class of compounds but failed to provide further guidance in the search for a potent and selective inhibitor. Microscale thermophoresis identified the following order of binding affinities for 3,4,5-trimethoxyphenol < 2,4,6-tribromophenol < pentabromophenol ⩽ TIP. Pentabromophenol and TIP inhibit myosin-6 ATPase activity in the low micromolar range, while the presence of either 100 μM 3,4,5-trimethoxyphenol or 2,4,6-tribromophenol has no effect on myosin-6 ATPase activity. The ATPase data shown in Fig. 3 suggest that relatively high concentrations of TIP are required for complete inhibition, while nearly complete inhibition of vesicle fusion occurs already at 5 μM. This apparent conflict may be related to differences in the rate limiting steps for ATP turnover and motor activity. Moreover, the observed IC_50_ value of 1.6 μM for the inhibition of secretion events suggests a TIP-induced shift in the ratio of strongly to weakly bound states in favor of strongly bound states.

In summary, our study shows that the human myosin motor domain construct M6-2R is a *bona fide* motor protein. The kinetic properties of human myosin-6 resemble in their key aspects those reported for the murine and porcine isoforms [25,44]. Differences such as 10-fold changes in actin and ADP affinities may conceivably be related to differences in the constructs used and their preparation [2,25,44]. Additionally, our results show that human myosin-6 can be specifically targeted by the organo halogenic compound TIP.

## Figures and Tables

**Table 1:** Summary of the kinetic parameters, rate and equilibrium constants of human myosin-6, murine myosin-6, and porcine myosin-6 TEDS-site mutants T406E and T406A.

Parameter^a^	Signal or calculation	*Hs* Myosin-6^a^^,^^b^	*Ss* Myosin-6 T406E^c^[25]	*Ss* Myosin-6 T406A^c^[25]	*Mm* Myosin-6 [44]
*k*_basal_ (s^−1^)	NADH assay	0.04 ± 0.004	<0.1	<0.1	0.18 ± 0.02 h
*k*_cat_ (s^−1^)	NADH assay	0.87 ± 0.01	8.3 ± 0.2	9.1 ± 0.8	2.77 ± 0.06^h^
*K*_app_ (μM)	NADH assay	0.58 ± 0.07	2.8 ± 0.3	17.6 ± 2.0	7.4 ± 0.7 h
*k*_cat_/*K*_app_ (μM^−1^ s^−1^)	NADH assay	1.50 ± 0.19	2.96 ± 0.4	0.51 ± 0.1	∼0.4
*K*_1_*k*_+2_ (μM^−1^ s^−1^)	d-mantATP	0.05 ± 0.01	0.27 ± 0.04	0.14 ± 0.04	0.06^i^
*k*_−2_ (s^−1^)	d-mantATP^d^	0.45 ± 0.01	3.9 ± 0.3	4.0 ± 0.3	n.d.
***K*_1_*k*_+2_** (μM^−1^ s^−1^)	Pyrene–actin	0.011 ± 0.0003	0.018 ± 0.0001	0.015 ± 0.000001	n.d.
***K*_1_*k*_+2_** (μM^−1^ s^−1^)	Light scattering	n.d.	n.d.	n.d.	0.013 i
***k*_+2_** (s^−1^)	Pyrene–actin	>140	>250	>250	n.d.
1/***K*_1_** (μM)	Pyrene–actin	∼12000	∼14000	∼14000	n.d.
*k*_+D_ (μM^−1^ s^−1^)	mantADP	0.14 ± 0.01	1.06 ± 0.10 (d-mantADP)	0.26 ± 0.04 (d-mantADP)	0.34 i
*k*_−D_ (s^−1^)	mantADP^e^	0.28 ± 0.01	n.d.	n.d.	n.d.
*k*_−D_ (s^−1^)	mantADP^f^	0.23 ± 0.01	6.4 ± 0.1	5.6 ± 0.2	5.7 i
*K*_D_ (μM)	*k*_−D_/*k*_+D_	2.0 ± 0.15	6.0 ± 0.6	21.5 ± 3.3	∼16.8
***k*_+AD_** (μM^−1^ s^−1^)	mantADP	0.20 ± 0.01	0.6 ± 0.10 (d-mantADP)	0.18 ± 0.03 (d-mantADP)	0.25 i
***k*_−AD_** (s^−1^)	mantADP^e^	0.16 ± 0.02	n.d.	n.d.	n.d.
***k*_−AD_** (s^−1^)	mantADP^f^	0.44 ± 0.01	5.6 ± 0.1	5.4 ± 0.2	6.8
***K*_i_** [Mg^2+^,free] (mM)	mantADP^e^	0.14 ± 0.06	n.d.	n.d.	n.d.
***K*_AD_** (μM)	***k*_−AD_**/***k*_+AD_**	0.8 ± 0.21	8.8 ± 1.4	30 ± 5.0	∼27.2
Coupling	***K*_AD_**/*K*_D_	∼0.76	∼1.46	∼1.39	∼1.6
***k*_+A_** (μM^−1^ s^−1^)	Pyrene–actin	2.4 ± 0.08^g^	5.4 ± 0.2	6.8 ± 0.2	n.d.
***k*_−A_** (s^−1^)	Pyrene–actin	∼0.014^g^	0.005 ± 0.004	0.004 ± 0.002	n.d.
***K*_A_** (nM)	***k*_−A_**/***k*_+A_**	∼5.8	0.9 ± 0.7	0.6 ± 0.3	n.d.
***k*_+DA_** (μM^−1^ s^−1^)	Pyrene–actin	0.26 ± 0.01^g^	0.9 ± 0.10	0.15 ± 0.1	n.d.
***k*_−DA_** (s^−1^)	Pyrene–actin	∼0.01^g^	0.06 ± 0.03	0.07 ± 0.03	n.d.
***K*_DA_** (nM)	***k*_−DA_**/**k_+DA_**	∼40.7	67 ± 34	47 ± 21	n.d.

a Reaction scheme and definitions are shown in Fig. 1.

b Experimental conditions: 20 mM MOPS (pH 7.0), 100 mM KCl, 5 mM MgCl_2_, 20 °C. The obtained stopped-flow transients are best described by mono-exponentials. In the case of the reaction between actomyosin and mantADP or d-mantADP, the obtained fluorescence transients were best-fit to a monoexponential plus lag-phase.

c Experimental conditions: 10 mM imidazole (pH 7.0), 50 mM KCl, 1 mM MgCl_2_, 1 mM EGTA, 1 mM DTT, 25 °C.

d From the intercept of the *k*_obs_ versus [d-mantATP] plot.

e From the intercept of the *k*_obs_ versus [mantADP] plot.

f From ATP chase experiment.

g Experimental conditions: 20 mM MOPS (pH 7.0), 5 mM MgCl_2_, 10 mM DTT, 20 °C.

h Experimental conditions: 30 mM KCl, 20 mM MOPS–KOH (pH 7.5), 3 mM MgCl_2_, 1 mM EGTA, 2 mM ATP, 25 °C.

i Experimental conditions: 30 mM KCl, 20 mM MOPS, 1 mM MgCl_2_, 1 mM EGTA, 5 mM β-mercaptoethanol (pH 7.5), 25 °C.
